# Transdermal delivery system to release phthalocyanine photosensitizers for the potential treatment of skin cancer with PDT

**DOI:** 10.55730/1300-0527.3665

**Published:** 2024-03-12

**Authors:** Meryem ÇAMUR DEMİR, Fatma KURŞUN BAYSAK, Caner Yahya BOYAR, Alihan TOKSOY, Fatih ALGI

**Affiliations:** 1 Department of Chemistry, Faculty of Science and Letters, Kırklareli University, Kırklareli, Turkiye; 2 Department of Biotechnology & ASUBTAM M. Bilmez BioNanoTech Lab., Aksaray University, Aksaray, Turkiye

**Keywords:** Transdermal drug delivery, phthalocyanines, SK-MEL-30, poly(vinyl alcohol)

## Abstract

This research aims to examine the transdermal release of water-soluble indium and zinc metallo phthalocyanine (InPc and ZnPc) compounds from the poly(vinyl alcohol) (PVA) membrane and the cytotoxicity effect of these Pcs on normal mouse fibroblasts (L929 fibroblast) and human melanoma (SK-MEL-30) cells. For this purpose, the effects of temperature, pH, drug concentration and membrane thickness on transdermal release were investigated in order to obtain the optimum transdermal release profile by preparing PVA membranes with different thicknesses and crosslinked by heat treatment. Optimum drug release was found to be 85.36% using 6 μm thick PVA membrane at 37 ± 0.5 °C, when upper cell pH 1.2 and lower cell pH 5.5, for 3 mg/mL InPc drug concentration. Under the same conditions, the drug release value for ZnPc was found to be 69.78%. In addition, in vitro studies were performed on L929 and SK-MEL-30 cells. under optimized drug (InPc and ZnPc) and membrane conditions. It was found that no significant cytotoxic effect was observed in L929 and SK-MEL-30 cells in the dark. Photodynamic tests were also carried out with InPc and ZnPc. The results show that cell viability decreases in SK-MEL-30 cells at concentrations of 10 μg/mL and above. In addition, while the InPc IC50 value was determined as 4.058 μg/mL, this value was determined as 11.574 μg/mL for ZnPc.

## 1. Introduction

Human skin resists drug distribution because it is a protective barrier that allows only one-way channeling, hinders the entry of external substances into the body, and resists the absorption of chemicals that come into touch with it [1,2]. A drug delivery system is a method that can control the distribution and release of the drug into cells, tissues and organs, efficiently delivering the drug to maximize therapeutic efficiency [3]. The transdermal drug delivery system is a method used to avoid the decisive limitations associated with oral drug delivery methods, by using the skin as a drug administration site, delivering biologically active agents to the systemic circulation through the blood vessels in the skin [4–7]. The transdermal drug delivery system has advantages such as comfortable and easy use, self-administration, compatibility with the patient, stable infusion and stable blood level, prevention of first pass metabolism, and no interaction with gastrointestinal fluids. However, it also has disadvantages such as high price, local irritation, lack of ionic drug release, low permeability, lack of rapid drug release, molecular size limitation (<500 Da) [8,9].

Various tools and approaches such as liquid jet injection, micro injection, micro needles, patches, gene gun and ultrasound methods are used to pass the drug through the skin. In the transdermal release method, patches are mostly used because they are cheap, safe and painless, as well as providing a superficial release [10].

Transdermal patches, which consist of three main layers: support layer, membrane layer and adhesive layer, have uncomplicated and easy use. Membrane structure is of great importance in controlling drug release. Polarity and size of the drug molecule, which cause agglomeration and low drug release, are the most important factors to be considered for the passage of the drug through the membrane [11].

Skin cancers, called melanoma, are usually diagnosed on sun-exposed skin areas such as the head, neck and hands, and are one of the most aggressive types of cancer with a very high mortality rate [12–14]. Surgical methods used in the treatment of melanoma cancer cause scars, tissue faults, longer recovery time and a growing risk of infection. However, chemotherapy also offers limited therapeutic options due to its important side effects and drug resistance. Therefore, noninvasive methods such as photodynamic therapy (PDT) are used for the treatment of skin cancer, which minimizes all these side effects, provides an optional treatment area, and does not have high effect, drug resistance or toxic side effects [15–17]. PDT is a promising treatment method in cancer treatment, which provides preferential destruction of tumor cells with photosensitizers, which can create the destruction of cancer cells once they are stimulated with laser radiation at a special wavelength with suitable energy density [18–21]. Phthalocyanine (Pc) compounds are used as drug or photosensitizer in PDT for the treatment of skin cancer cells [22–24].

Monton et al. examined the transdermal release of propranolol hydrochloride using a polyvinyl alcohol-graft-lactic acid (PVA-g-LA) membrane and presented the cumulative release of the drug as 61.94% ± 8.03% [25]. In recent years, Pc-derived drugs have started to replace drugs such as metoclopramide, amoxicillin and doxorubicin, whose transdermal release has been studied especially in skin cancer treatment [11,26–32].

**Figure 1:**
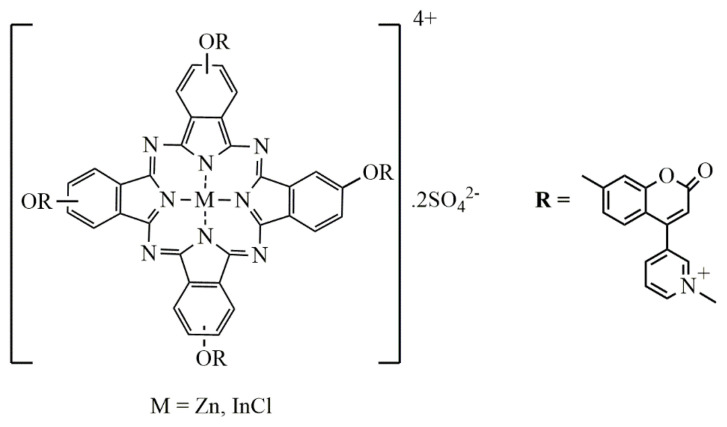
Water-soluble InPc and ZnPc compounds.

In this study, transdermal release from the poly(vinyl alcohol) (PVA) membrane of water-soluble indium and zinc metallo Pc compounds **(**InPc and ZnPc) (Figure 1) which synthesized in previous study [33] was investigated. According to this study, InPc and ZnPc produced the highest singlet oxygen quantum yields (unsusbstituted ZnPc was used as standard) which these results indicate the water soluble InPc and ZnPc compounds may be a new promising photosensitizers for PDT. Pcs are larger molecules than commercial drugs, so their passage through the membrane is more difficult than commercial drugs. It was evaluated that PVA would be effective in the controlled release of Pc’s thanks to its high hydrophilicity and elasticity.

The effects of temperature, pH, drug concentration and membrane thickness on InPc and ZnPc release were investigated. Additionally, MTT testing was performed on normal mouse fibroblasts (L929 fibroblast) and human melanoma (SK-MEL-30) cells under optimized drug and membrane conditions.

## 2. Materials and methods

### 2.1. Materials

Synthesis of InPc and ZnPc compounds and their photophysical and photochemical properties have been given in the previous publication [33]. PVA with a number average molecular weight of 89,000 to 98,000 and a 98%–99% degree of hydrolysis was obtained from Merck and used as received. Triton X-100 was purchased from Merck. SK-MEL-30 and L929 cells were obtained from the Institute of ŞAP, Ankara, Türkiye.

### 2.2. Preparation of PVA membranes

It was obtained by mixing 2% PVA solutions in distilled water at 85 °C. Different volumes of the prepared PVA solution were poured into petri dishes and dried in an oven until complete dryness. The dried membranes were taken from petri dishes and their thicknesses were measured using a micrometer (Dasqua Digital Micrometer (0.001 mm Precision)). Membranes dried to full dryness were thermally crosslinked in an oven at 150 °C for 1 h.

### 2.3. Transdermal release process

The transdermal drug delivery study was carried out in a Franz diffusion cell using cross-linked membranes in a constant temperature adjustable water bath. The lower component of the Franz diffusion cell was supplied with a buffer solution at the pH to be studied and the upper component was supplied with 2 mg/mL of drug solution prepared by dissolving 5 mL in the pH buffer to be studied. 0.5 mL of solution was taken from the lower component of the Franz diffusion cell at certain times, the total volume was completed to 3.5 mL, and the drug concentration that passed through the membrane was calculated by reading it in the UV-Vis device under the conditions given in Table. Instead of 0.5 mL of solution taken from the lower component of the Franz diffusion cell, the buffer solution at the working pH was added [34].

### 2.4. Cell culture

Cells were free of mycoplasma, bacteria, and fungi. SK-MEL-30 and L929 cells were grown in Dulbecco’s modified Eagle’s medium (DMEM) with 10% fetal bovine serum (FBS), 100 IU/mL penicillin, and 100 mg/mL streptomycin, and incubated at 37 ± 0.5 °C in a humidified incubator containing 5% CO_2_. The cells were extracted for use in following experiments by trypsinizing (trypsin 0.025%, EDTA 0.02%), followed by a PBS wash.

Normal and malignant cells (1×10^4^) were planted in 96-well plates using fresh DMEM culture media, and they were then cultured for 24 h at 37 ± 0.5 °C under 5% CO_2_. InPc and ZnPc were dissolved in DMEM then added to fresh cell culture medium in various concentrations (0, 5, 10, 25, and 50 mg/mL), and the cells were then incubated. The cells were cleaned with PBS solution after a predetermined amount of incubation (24 h). The viability of the cells was then evaluated using the Thiazolyl blue tetrazolium bromide (MTT) assay. Each experiment was carried out five times, and the corresponding data are shown as the mean ± SD.

### 2.5. In vitro photodynamic treatment

Several concentrations of InPc and ZnPc (0, 1, 5, 10, 25, and 50 mg/mL) were incubated with the malignant cells (1×10^4^) for 12 h. Following a PBS wash, the cells were exposed to a red light source (660 nm; power density: 6,2 J/cm^2^) for 60 min. The MTT test was then used to determine the viability of the cells. Each experiment was carried out five times, and the data collected is shown as the mean ± SD.

### 2.6. MTT assay

To test the cell viability, thiazolyl blue tetrazolium bromide (MTT) assay was utilized. MTT is converted into an insoluble formazan by living cells. Dimethyl sulfoxide (DMSO) can dissolve the resultant formazan, and absorption spectroscopy is used to quantify its concentration [35]. An ELISA reader (ChroMate 4300 ELISA Reader) is used to measure the absorbance at 492 nm. Each experiment was repeated 5 times, and data are represented as the mean±SD.

## 3. Results and discussion

### 3.1. Effects of membrane thickness and pH

In order to examine the effect of membrane thickness on drug release, PVA membranes with different thicknesses were investigated at 25 ± 0.5 °C, at 2 mg/mL water-soluble InPc drug concentration, in pH 1.2, 5.5 and 7.4 buffers. The results of membrane thickness and using pH 1.2, 5.5 and 7.4 buffer are presented in Figure 2a, b, and c, respectively. As seen in all figures, the amount of drug diffused from the unit area decreased with the increase in membrane thickness, and thus the drug release values decreased with the increase in thickness. This can be explained by the increase in its thickness, the more difficult the diffusion of the drug to the membrane, and the decrease in the amount of drug passing through the membrane. The maximum drug release value was found to be 12.29% in the PVA membrane with a thickness of 6 μm in pH 5.5 buffer [24].

In Figure 2a, b, and c, it is seen that InPc release changes depending on pH. In an acidic medium (pH: 1.2), the hydroxyl groups of PVA on the membrane are protonated and the membrane becomes positively charged. Due to the repulsion between the positively charged membrane and drug molecules, drug release from the membrane is reduced. Therefore, the amount of InPc released from the PVA membrane at low pH is lower than at other pHs. It is thought that the strong hydrogen bonds formed between the membrane and the buffer solution at high pH do not allow the drug to pass through [33,35].

### 3.2. Effect of temperature

**Figure 3:**
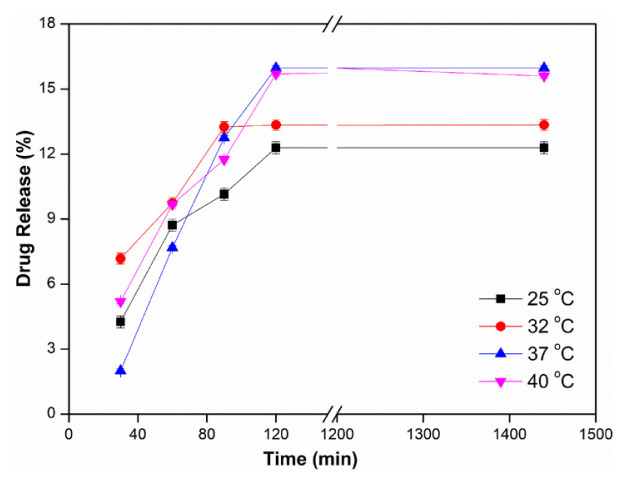
Effect of temperature (pH: 5.5, Thickness: 6 μm, InPc: 2 mg/mL).

The effect of temperature (25, 32, 37, and 40 °C) on drug release was investigated in pH 5.5 buffer, at 2 mg/mL water-soluble InPc drug concentration, using a 6 μm thick PVA membrane. In Figure 3, it is seen that the amount of drug release increases with the increase in temperature. The maximum drug release value was found to be 15.97% at 37 ± 0.5 °C and there was no change at 40 ± 0.5°C. PVA is a hydrophilic membrane due to the presence of –OH groups in its structure. As the temperature increases, the thermal movements of the polymer chains in the amorphous regions of the PVA membrane increase, thus increasing the free volume in the membrane structure and thus increasing the diffusion of the drug to the membrane. However, with the increase in temperature, the mobility of the drug also increases and the H-bond interactions between the drug and PVA membrane weaken, and it becomes easier for the drug to drift from the membrane. Similar results are also found in the literature [36,37]. Rossetti et al. synthesized a phthalocyanine derivative, zinc phthalocyanine tetrasulfonate (ZnPcSO_4_), for use in photodynamic therapy. They were released at 37 ± 0.5 °C in a microemulsion medium consisting of Span80/Tween 80/canola oil/propylene glycol (PG)/water mixture, which makes it difficult for ZnPcSO_4_ to penetrate the skin and prevents aggregation. As a result of their studies, they confirmed that the microemulsion medium increased both the penetration and biodistribution of ZnPcSO_4_ into the skin [37].

### 3.3. Influence of donor compartment pH

**Figure 4:**
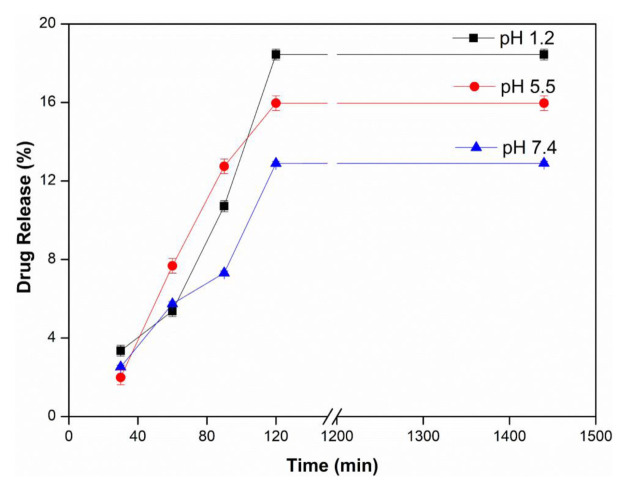
Effect of upper cell pH (Lower Cell pH: 5.5, T: 37 ± 0.5 °C, Thickness: 6 μm, InPc: 2 mg/mL).

The effect of upper cell pH on drug release was investigated using a 6 μm thick PVA membrane at 37 ± 0.5 °C, pH 5.5 buffer, 2 mg/mL water-soluble InPc drug concentration. In Figure 4, it is seen that the amount of drug release decreases with the increase of upper cell pH. The maximum drug release value was found to be 18,45% at pH 1.2. High drug diffusion through the membrane was obtained at pH 1.2. At pH 1.2, it is expected that the electrostatic repulsion of the cationic groups of InPc will increase and the drug permeation through the PVA membrane will increase. Similar findings were reported by Geyik and Işıklan in their study of 5-Fluorouracil release from κ-carrageenan-based nanospheres. They reported that since 5-Fluorouracil is an acidic drug (pKa ~ 8), its solubility also depends on pH, and they achieved a faster release rate thanks to the repulsive interactions of the drug with the positively charged surface shell [38].

### 3.4. Effect of drug concentration

**Figure 5:**
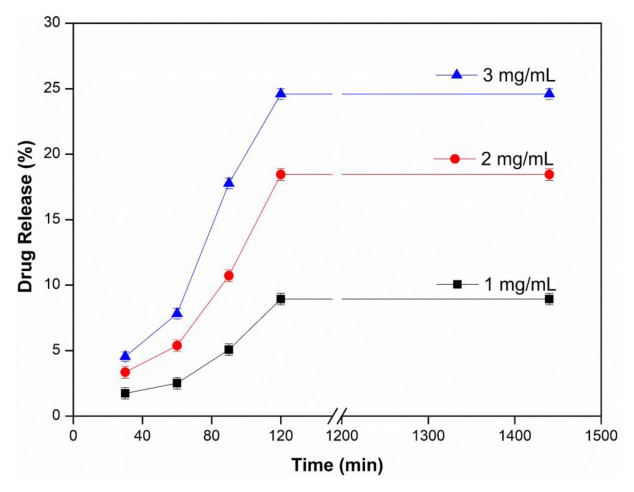
Effect of drug concentration (Upper Cell pH: 1.2, Lower Cell pH: 5.5, T: 37 ± 0.5 °C, Thickness: 6 μm).

The effect of drug concentration on drug release was investigated by changing the drug concentration in the range of 1–3 mg/mL in the upper cell pH 1.2 and lower cell pH 5.5 buffer at 37 ± 0.5 °C using a 6 μm thick PVA membrane. In Figure 5, it is seen that the amount of drug release increases with the increase in the amount of drug. The maximum drug release value was found to be 24.59% at 3 mg/mL water-soluble InPc concentration. It was thought that the % drug release increased as the amount of drug diffused along the membrane and therefore the amount of drug passing through the membrane would increase with the increase in the amount of drug. Denkbaş et al. reported that the release of 5-Fluorouracil from chitosan microspheres increased when the initial drug concentration was increased, as expected [39].

### 3.5. Effect of Triton X-100 amount

**Figure 6:**
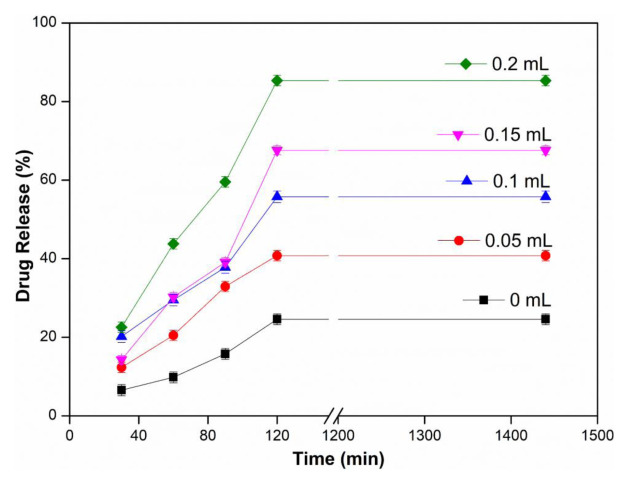
Effect of Triton X-100 amount (InPc: 3 mg/mL, Upper Cell pH: 1.2, Lower Cell pH: 5.5, T: 37 ± 0.5 °C, Thickness: 6 μm).

The effect of Triton X-100 amount on drug release was investigated at 37 ± 0.5 °C, in upper cell pH 1.2 and lower cell pH 5.5 buffer, at 3 mg/mL water-soluble InPc drug concentration, using a 6 μm thick PVA membrane. In Figure 6, it is seen that the percentage of drug release increases with the increase in the amount of Triton X-100. The maximum drug release value was found to be 85.36% when 0.2 mL of Triton X-100 was added. In drug release studies, it was aimed to increase drug transfer by using Triton X-100. Since Triton X-100 is a surfactant, it is expected to act in the direction of increasing the permeability of the drug. While the drug release value was 24.59% under the same experimental conditions without using Triton X-100, it increased almost 3.5 times and reached 85.36% when Triton X-100 was used. Charoo et al. investigated the penetration enhancing potential of tulsi and turpentine oil on transdermal delivery of flurbiprofen, a potent nonsteroidal antiinflammatory agent. It was observed that there was an additive effect on the skin permeation rate of flurbiprofen in the binary solvent mixture (propylene glycol (PG): isopropyl alcohol (IPA)) when tulsi and turpentine oil were added separately to the optimized cosolvent mixture [40].

### 3.6. ZnPc release profile at optimum conditions

**Figure 7:**
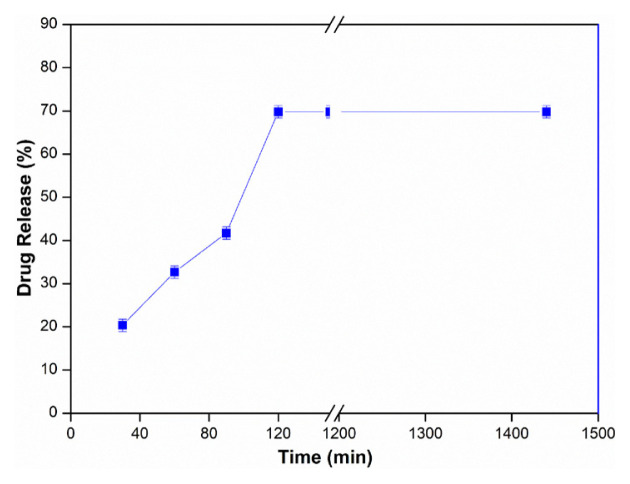
ZnPc release profile under optimum conditions (Triton X-100: 0.2 mL, ZnPc: 3 mg/mL, Upper Cell pH: 1.2, Lower Cell pH: 5.5, T: 37 ± 0.5 °C, Thickness: 6 μm).

Release profile of water-soluble ZnPc under optimum conditions, at 37 ± 0.5 °C, in upper cell pH 1.2 and lower cell pH 5.5 buffer, Triton X-100 0.2 mL, 3 mg/mL water-soluble ZnPc drug concentration, 6 μm thickness were investigated using a PVA membrane with a high density and is presented in Figure 7. The maximum drug release value was found to be 69.78%.

### 3.7. Cytotoxicity of InPc and ZnPc on L929 cells

**Figure 8:**
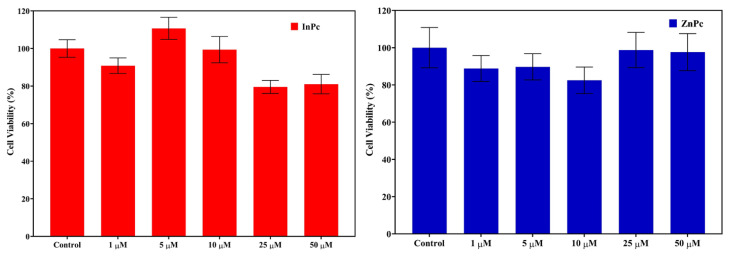
The viability of L929 fibroblast cells after treatment with different concentrations of (a) InPc, and (b) ZnPc for 24 h in the dark. The results are expressed as mean ± SD (n = 5), *p < 0.05 (compared with PBS as control).

The cytotoxicity of InPc and ZnPc were investigated to reveal the biocompatibility of these compounds. Different concentrations (0, 5, 10, 25, and 50 μg/mL) were used to evaluate the cytotoxicity of InPc and ZnPc towards dermal fibroblast cell line (L929) cells after 24 h incubations. The results were depicted in Figure 8. It is noteworthy that InPc and ZnPc did not induce any significant change on the cell viability of L929 cells. However, at higher concentration (25 and 50 μg/mL) after 24 h, the viability of the cells was decreased up to 80% for InPc (Figure 8).

### 3.8. In vitro imaging and photodynamic action of InPc and ZnPc

**Figure 9:**
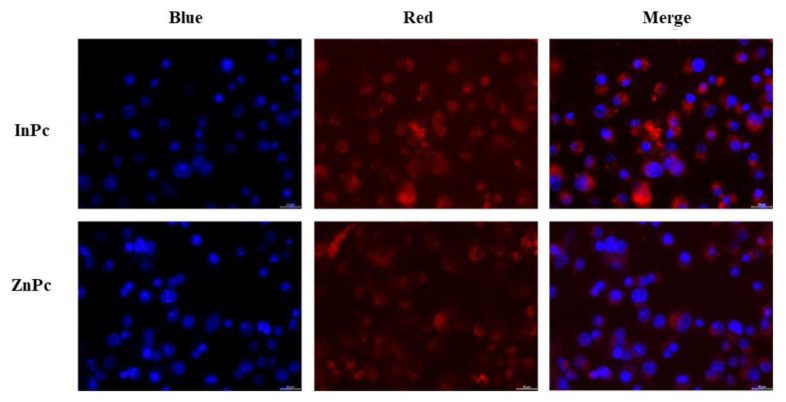
Nuclear morphology of SK-MEL-30 cells incubated with InPc and ZnPc for 12 h and DAPI staining. Scale bar = 20 μm. Filter λ_ex_: 620/60 nm.

We carried out fluorescence microscopy experiments to visualize the cellular internalization of InPc and ZnPc into SK-MEL-30 cells. To this end, SK-MEL-30 cells were incubated with InPc and ZnPc (10 μg/mL) for 12 h. We found that both InPc and ZnPc were permeable into SK-MEL-30 cells (Figure 9, red channel). The data confirmed that InPc and ZnPc enabled live-cell imaging of cancerous cells.

**Figure 10:**
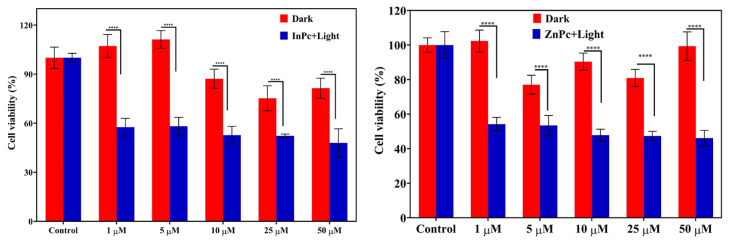
The viability of SK-MEL-30 cells after treatment with different concentrations of (a) InPc, and (b) ZnPc in the dark (red bars) or after illumination (blue bars) for 60 min. The results are expressed as mean ± SD (n = 5), ****p < 0.001 (compared with control).

To investigate the (photo)cytotoxicity of InPc and ZnPc, we carried out in vitro (photo)cytotoxicity tests using SK-MEL-30 cells. We used different concentrations of InPc and ZnPc (0, 5, 10, 25, and 50 μg/mL) for incubation (24 h) and the samples were illuminated with red light. Since the absorption maxima of InPc and ZnPc were around 650–700 nm, red light (660 nm) was chosen for PDT studies. It should be emphasized that we excluded the illumination step in the control experiments. Finally, we evaluated the cell viability using MTT assay. Our results revealed that both InPc and ZnPc exhibited significant photocytotoxicity (blue bars) compared to control groups (red bars) (Figure 10).

**Figure 11:**
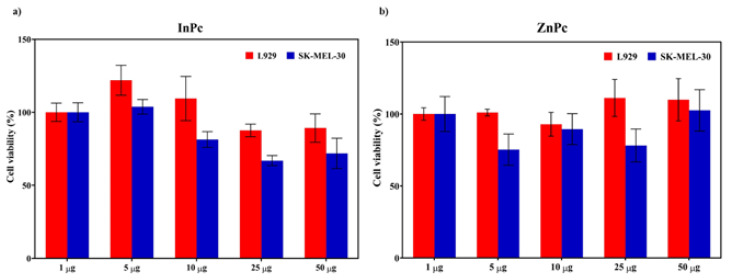
Dark toxicity of InPc and ZnPc for L929 fibroblast cells and SK-MEL-30 cell line.

We also compared the dark toxicity of InPc and ZnPc for both L929 fibroblast cells and SK-MEL-30 cells. Figure 11 depicts that InPc and ZnPc are more toxic (see blue bars) to cancer cells when compared to L929 cells.

**Figure 12:**
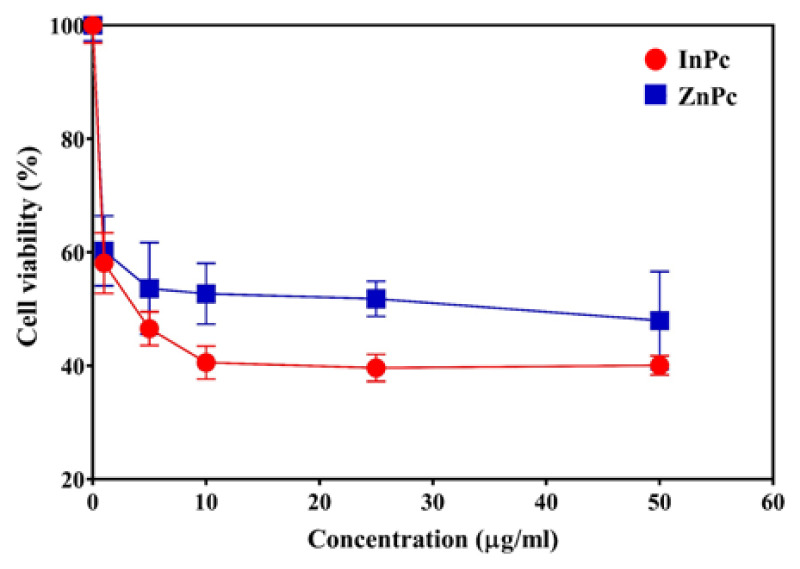
The half maximal inhibitory concentrations (IC50) for InPc and ZnPc in SK-MEL-30 cell line.

The half maximal inhibitory concentrations (IC_50_) for InPc and ZnPc were found to be 4.058 μg/mL and 11.574 μg/mL, respectively (Figure 12). It is noteworthy that both InPc and ZnPc can be used as photosensitizers for in vitro studies.

The singlet oxygen quantum yields of ZnPc and InPc were reported as 0.06 and 0.08, respectively (in water) [33]. Obviously, the singlet oxygen quantum yield of InPc was slightly higher than ZnPc. Consequently, IC_50_ value of InPc was found to be lower than that of ZnPc. On the other hand, the cell viability rates obtained with ZnPc appeared to be lower than InPc.

## 4. Conclusion

This study focused on the use of water soluble phthalocyanines in drug delivery systems. Release profiles of water-soluble InPc and ZnPc were investigated under optimum conditions: 37 ± 0.5 °C temperature, in upper cell pH 1.2 and lower cell pH 5.5 buffer, Triton X-100 0.2 mL, 3 mg/mL water-soluble drug concentration and 6 μm thickness a PVA membrane. Maximum drug release values of InPc and ZnPc were found to be 85.36% and 69.78%, respectively.

We found that InPc and ZnPc did not induce cytotoxicity against L929 cell lines. However, it should be noted that InPc and ZnPc are more toxic to cancer cells when compared to L929 cells. Moreover, we showed that InPc and ZnPc can be used for in vitro fluorescence imaging of cancer cells. Finally, InPc and ZnPc were significantly more photocytotoxic to cancer cells compared to the dark control groups. IC_50_ values for InPc and ZnPc were 4.058 mg/mL and 11.574 mg/mL, respectively.

Transdermal patches of InPc and ZnPc to be prepared with PVA membrane can be recommended to be used especially for the treatment of the cancerous area on the skin surface, since they are released from the PVA membrane in a short time, as well as not showing cytotoxic effects. It is suggested the release profile and cytotoxicity results of InPc and ZnPc is compatible with PDT and these Pcs have potential for the treatment of skin cancer. On the other hand, the mechanisms and other factors during PDT are many complicated and not exactly known yet; and so more works are necessary in this regard.

## Figures and Tables

**Figure 2a:**
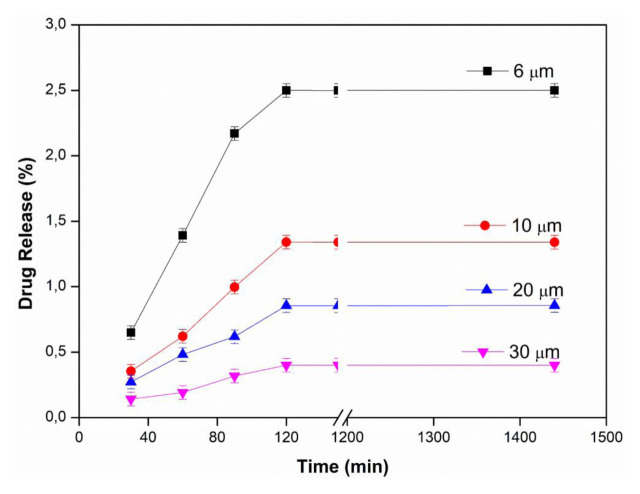
Effect of membrane thickness at pH: 1.2 (T: 25 ± 0.5 °C, InPc: 2 mg/mL).

**Figure 2b:**
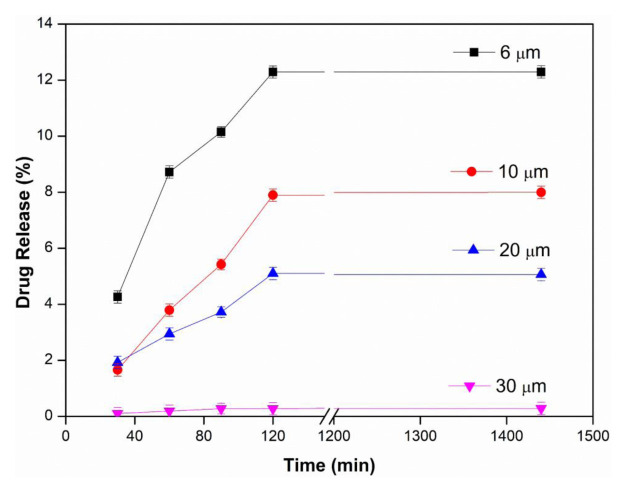
Effect of membrane thickness at pH: 5.5 (T: 25 ± 0.5 °C, InPc: 2 mg/mL).

**Figure 2c:**
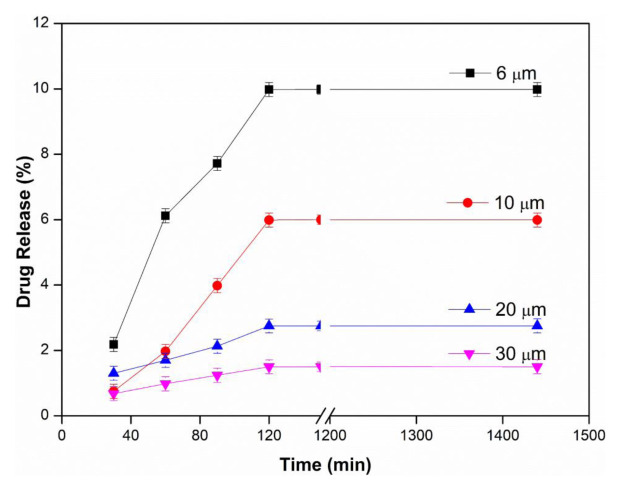
Effect of membrane thickness at pH: 7.4 (T: 25 ± 0.5 °C, InPc: 2 mg/mL).

**Table:** Experimental parameters for phthalocyanines in UV-Vis (n = 5).

Wavelength, nm	680 ± 2
Linear range, mol L^−1^	2.10^−7^−1.2.10^−−6^
Linearity	y = 30229x − 0.0163
Regression coefficient, R^2^	0.9972
The limit of detection, LOD, mol L^−1^	0.0025
The limit of quantification, LOQ, mol L^−1^	0.0061
The relative standard deviation (RSD), %	1.2–5.3
Recovery, %	97.1 ± 6.4
